# Targeting mitochondria as an anticancer strategy

**DOI:** 10.1186/s40880-019-0412-6

**Published:** 2019-10-25

**Authors:** Lanfeng Dong, Jiri Neuzil

**Affiliations:** School of Medical Science, Griffith University, Southport, QLD 4222 Australia

## Mitochondrial metabolism and cancer development

Mitochondria are organelles controlling adenosine triphosphate (ATP) generation, redox homeostasis, metabolic signaling, and apoptotic pathways. Although glycolysis was traditionally considered as the major source of energy in cancer cells, in-line with the so-called “Warburg effect”, mitochondria have been recognized to play a key role in oncogenesis [1]. Cancer cells uniquely reprogram their cellular activities to support their rapid proliferation and migration, as well as to counteract metabolic and genotoxic stress during cancer progression [2]. Further, mitochondria can switch their metabolic phenotypes to meet the challenges of high energy demand and macromolecular synthesis [3]. Thus, cancer mitochondria have the ability to flexibly switching between glycolysis and oxidative phosphorylation (OXPHOS) for their survival. The electron transport chain (ETC) function is pivotal for mitochondrial respiration, which is also needed for dihydroorotate dehydrogenase (DHODH) activity that is essential for de novo pyrimidine synthesis [4]. Recent researches have demonstrated that cancer cells devoid of mitochondrial DNA (mtDNA) lack their tumorigenic potential, and they re-gain this ability by acquiring healthy mtDNA from the host stromal cells via horizontal transfer of whole mitochondria [5, 6] for recovery of the respiratory function. Functionally, respiration propels DHODH activity for pyrimidine biosynthesis [7]. Therefore, targeting mitochondria holds great potential for anticancer strategy with high therapeutic opportunities.

## Targeting mitochondria as a therapeutic anticancer approach

Multiple strategies have been developed to target mitochondria for cancer therapies including agents that target electron transport chain and the OXPHOS function, glycolysis, the tricarboxylic acid (TCA) cycle, apoptotic pathways, reactive oxygen species (ROS) homeostasis, the permeability transition pore complex, mitochondrial DNA as well as DHODH-linked pyrimidine synthesis [8, 9]. In this research highlights, we demonstrate some of the most relevant mitochondrial targets in cancer therapy.

## Targeting mitochondrial metabolism

### (i) Targeting ETC

Functional ETC supports OXPHOS activity and adenosine triphosphate (ATP) generation that is essential for tumorigenesis. Many ETC inhibitors, such as metformin, tamoxifen, α-tocopheryl succinate (α-TOS) and 3-bromopyruvate (3BP), act via disrupting the function of respiratory complexes of the ETC and inducing high levels of ROS to kill cancer cells [8, 9]. A novel approach of selective targeting of cancer mitochondria by tagging a cationic triphenylphosphonium (TPP^+^) group to anticancer compounds (e.g., α-TOS, tamoxifen and metformin) is considered as a mitochondrial-targeted therapy, delivering drugs preferentially into cancer cell mitochondria based on their higher transmembrane potential to trigger mitochondria-dependent apoptosis via rapid generation of ROS [9, 10]. Both MitoVES (mitochondrially targeted vitamin E succinate targeting complex II) and MitoTAM (mitochondrially targeted tamoxifen targeting complex I) have been prepared by tagging TPP^+^ to parental compounds efficiently kills colorectal, lung and breast cancer cells and inhibits tumor growth by interfering with complex I-/complex II-dependent respiration without systemic toxicity [11, 12].

### (ii) Targeting glycolysis and OXPHOS

The glycolysis metabolic pathway directly affects mitochondrial function by providing key metabolic intermediates, such as pyruvate, for mitochondrial metabolism. Moreover, the ability of malignant cells to flexibly switching between glycolysis and oxidative phosphorylation appears to play a major role in multiple modes of resistance to oncogenic inhibition [1, 8]. Therefore, agents that target both glycolysis and OXPHOS function hold promise as an ideal anticancer therapeutic approach. Mitochondria-targeted therapeutics in combination with glycolytic inhibitors synergistically suppress tumor cell proliferation [9]. Hexokinase II (HKII) is a major isoform of the enzyme overexpressed in cancer cells and plays an important role in maintaining glycolytic activity. It also binds to the voltage-dependent anion channel (VDAC) on the mitochondrial outer membrane. As such, inhibition of HKII will not only inhibit glycolysis but also suppresses the anti-apoptotic effects of the HKII–VDAC interaction. Several hexokinase inhibitors have been found to suppress cancer growth. FV-429 is a synthetic flavone with potent activity to induce apoptosis in cancer cells by inhibition of glycolysis via suppression of HKII and impairing mitochondrial function via interfering with the HKII–VDAC interaction, leading to activation of mitochondrial-mediated apoptosis. Metformin, a drug commonly used to treat diabetes, can suppress multiple types of cancers [13, 14]. Recent report showed that metformin inhibits HKII in lung carcinoma cells to decrease glucose uptake and phosphorylation. Combining metformin with 2-deoxyglucose (2-DG), a glycolysis inhibitor, depleted ATP in a synergistic manner and showed a strong synergy for the combined therapeutic effect in pancreatic cancer cells. The mitochondria-targeted drug, mitochondria-targeted carboxy-proxyl (Mito-CP) in combination with 2-DG led to significant tumor regression, suggesting that dual targeting of mitochondrial bioenergetic metabolism and glycolytic inhibitors may offer a promising chemotherapeutic strategy [15].

### (iii) Targeting the TCA cycle

The TCA cycle is a source of electrons that feed into the ETC to drive the electrochemical proton gradient required for ATP generation. Isocitrate dehydrogenases 1 and 2 (IDH1, IDH2) catalyzes the conversion of isocitrate to α- ketoglutarate, playing a critical role in tumorigenesis [9]. Mutations in IDH1 and IDH2 have been found in different human cancers [16] that render them as promising targets for anticancer therapy. Inhibitors of IDHs such as AGI-5198, AGI-6780, AG-120, AG-221, 3BP, and dichloroacetate possess high anticancer potential in a broad range of cancer types [8, 17].

## Targeting apoptotic pathways and ROS homeostasis

### (i) Targeting Bcl-2 family proteins

Bcl-2, Bcl-xL, Bax, and Bak are important in the intrinsic apoptotic pathway. Venetoclax, currently approved for use in patients with chronic lymphocytic leukemia [18], navitoclax, TW-37, GX15-070 and BM-1197, are Bcl-2 or Bcl-xL inhibitors with anticancer activity in a broad range of cancer types [8]. Compounds such as Gossypol, Navitoclax, ABT-737 and α-TOS act as mimetics of the Bcl-2 homology-3 domains to kill cancer cells through the activation of post-mitochondrial apoptotic signaling [17].

### (ii) Targeting redox-regulating enzymes and ROS production

Electron transport chain is the major site of ROS production, and high level of ROS released due to interference with the ECT complexes cause cellular damage. Oxymatrine was reported to efficiently kill human melanoma cells by generating high levels of ROS. Capsaicin, casticin, and myricetin display anticancer activity by increasing ROS generation, leading to the disruption of mitochondrial transmembrane potential in cancer cells [8]. Promoting mitochondrial ROS production to induce cancer cell death may enhance the activity of chemotherapy [15]. By coupling triphenylamine (TPA) with the fluorophore BODIPY, a novel mitochondrial-targeted fluorescent probe BODIPY-TPA was shown to induce apoptosis in gastric cancer via disruption of the mitochondrial redox balance and ROS accumulation [19].

In summary, mitochondria play a key role in cell survival and apoptosis. Mitochondrial respiration supports ATP production and is also essential for tumorigenesis. Targeting mitochondrial metabolism presents a new concept to effective cancer therapeutics.

## Data Availability

Not applicable.

## Abbreviations

ATP: adenosine triphosphate
OXPHOS: oxidative phosphorylation
ETC: electron transport chain
DHODH: dihydroorotate dehydrogenase
TCA cycle: tricarboxylic acid cycle
ROS: reactive oxygen species
α-TOS: α-tocopheryl succinate
3BP: 3-bromopyruvate
TPP^+^: cationic triphenylphosphonium
MitoVES: mitochondrially targeted vitamin E succinate
MitoTAM: mitochondrially targeted tamoxifen
HKII: hexokinase II
VDAC: voltage-dependent anion channel
2-DG: 2-deoxyglucose
IDH1/IDH2: isocitrate dehydrogenases1/2
TPA: triphenylamine
